# Adjacent Lumbar Disc Herniation after Lumbar Short Spinal Fusion

**DOI:** 10.1155/2014/456940

**Published:** 2014-09-07

**Authors:** Koshi Ninomiya, Koichi Iwatsuki, Yu-ichiro Ohnishi, Toshika Ohkawa, Toshiki Yoshimine

**Affiliations:** Department of Neurosurgery, Osaka University Graduate School of Medicine, 2-2 Yamadaoka, Suita, Osaka 565-0871, Japan

## Abstract

A 70-year-old outpatient presented with a chief complaint of sudden left leg motor weakness and sensory disturbance. He had undergone L4/5 posterior interbody fusion with L3–5 posterior fusions for spondylolisthesis 3 years prior, and the screws were removed 1 year later. He has been followed up for 3 years, and there had been no adjacent segment problems before this presentation. Lumbar magnetic resonance imaging (MRI) showed a large L2/3 disc hernia descending to the L3/4 level. Compared to the initial MRI, this hernia occurred in an “intact” disc among multilevel severely degenerated discs. Right leg paresis and bladder dysfunction appeared a few days after admission. Microscopic lumbar disc herniotomy was performed. The right leg motor weakness improved just after the operation, but the moderate left leg motor weakness and difficulty in urination persisted.

## 1. Introduction

The incidence of adjacent segment disease after spinal fusion is reportedly 5.2–49% [1]. Among them is adjacent disc degeneration, which may be due to increased stress adjacent to fusion. However, to the best of our knowledge, only three cases of thoracic disc herniation after posterior fusion have been reported [2–4]. Here, we present a case of sudden lumbar huge disc herniation with acute paraplegia after posterior short fusion without any adjacent segment degeneration just before the herniation occurred.

## 2. Case Presentation

A 70-year-old man came to our outpatient department with sudden left leg motor weakness. He had undergone L4/5 posterior interbody fusion with L3–5 posterior fusion for spondylolisthesis 3 years previously, and the screws were removed 1 year later. He had slight right leg motor weakness but could walk by himself for the 3 following years.

**Figure 1:**
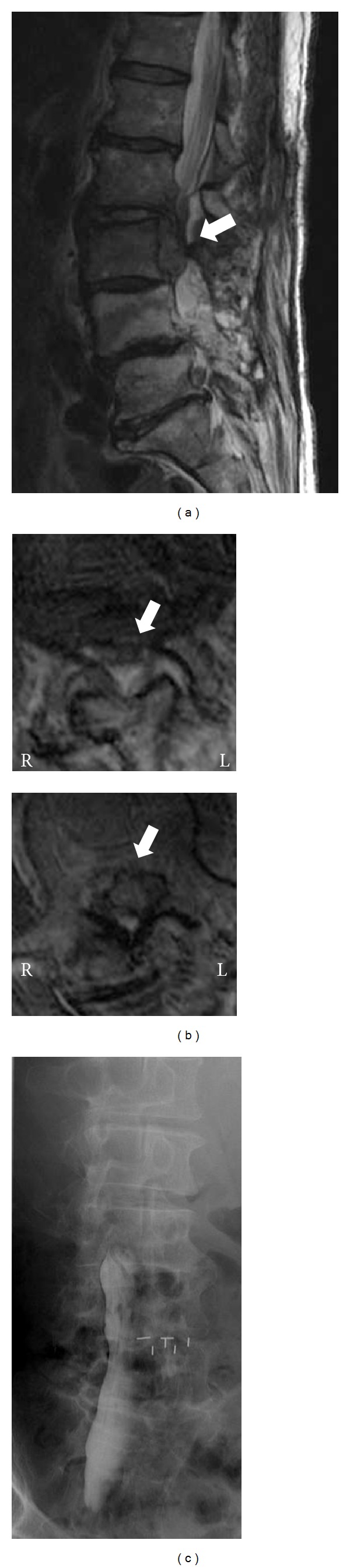
(a) Sagittal T2-weighted magnetic resonance image at the time of admission demonstrating a large descending disc hernia from the L2/3 to L3 levels. (b) Axial T2-weighted image of the L2/3 level (upper) and L3 vertebra level (lower) showing a hernia (arrow) compressing the spinal cord posterolaterally. (c) Myelogram obtained in a head-lowering prone position showing obstruction at the L3 vertebra level.

Lumbar magnetic resonance imaging (MRI) showed a huge mass located behind the L3 vertebral body (Figures 1(a) and 1(b)). Its intensity was almost equivalent to that of the L2/3 disc. A descending hernia from the L2/3 disc that severely compressed the dural sac anteriorly was suspected.

**Figure 2:**
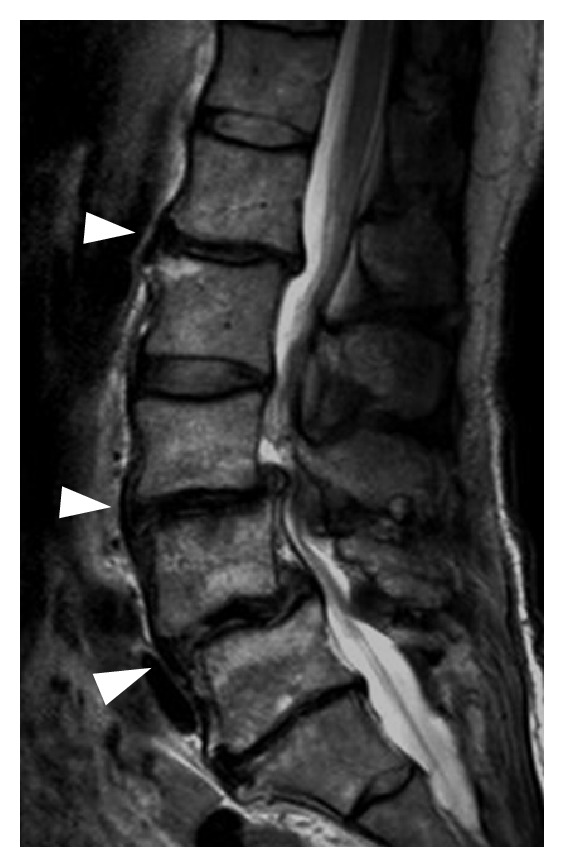
Sagittal T2-weighted magnetic resonance image before the spinal fusion operation showing severe disc degeneration (arrowhead) at the L1/2, L3/4, and L4/5 levels.

**Figure 3:**
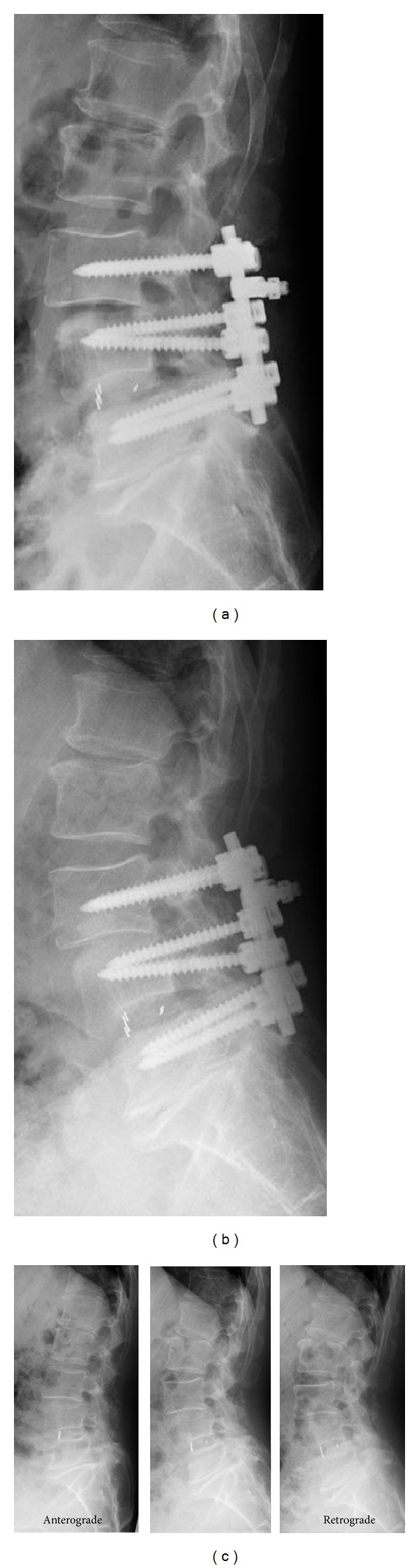
Plain lumbar radiographs taken just after the fusion operation (a), 1 year after the operation (b), and 2 years after the operation (c). There was slight retrolisthesis and decreased posterior disc height on L2/3 on 1-year (b) and 2-year follow-up (c), but with no definite instability (c).

On the initial lumbar MRI taken prior to the fusion operation, severe degeneration was seen in the L1/2, L3/4, and L4/5 discs (Figure 2). According to Pfirrmann classification [5], they were grade V compared to L2/3, which was grade II. Follow-up lumbar radiographs demonstrated slight change in the adjacent posterior disc height, but there was no change in the adjacent vertebral body height or definite adjacent instability before this presentation (Figure 3).

On the day of admission, he showed manual muscle test (MMT) 1 motor weakness of the left iliopsoas and left quadriceps and MMT 3 of the left hamstrings. Hypesthesia was noted from the anterior part of the left thigh to the inner lower leg. Myelography showed an obstruction of the contrast medium at the L3 level (Figure 1(c)).

**Figure 4:**
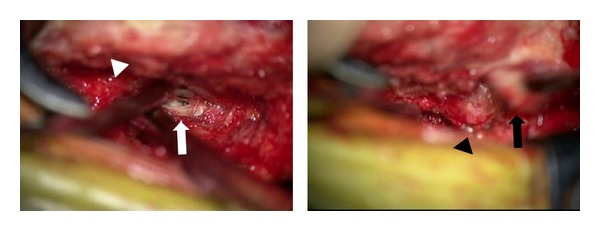
Intraoperative view showing the left L3 nerve root (white arrowhead), left L4 nerve root (black arrowhead), disc hernia (white arrow), and removed hernia (black arrow).

Three days after admission, severe right leg motor weakness and incomplete urinary retention appeared. Therefore, a disc hernia removal operation was performed. Under general anesthesia, the patient was laid in the prone position. A midline skin incision was made from L2 to the L4 spinous process. After dissection of the paraspinal muscles, a hemilaminectomy of the inferior half of the L2 and L3 laminae was performed. Below the posterior longitudinal ligament, a sequential mass was observed from the L2/3 disc to the L3 vertebral body level. The mass was comparatively hard, and a piecemeal removal was performed. Left L3 and L4 nerve root decompression was confirmed, and the wound was closed in the usual manner (Figure 4).

After the operation, the patient's right lower extremity paresis resolved quickly. However, the moderate left leg motor weakness and urinary disturbance persisted, so he was transferred to a rehabilitation hospital.

## 3. Discussion

Proximal adjacent segment degeneration or disc degeneration after instrumented spinal fusion has been widely reported [6–8]. However, to our knowledge, there have been only three reports of disc hernia after spinal fusion [2–4]. All the cases occurred in the thoracic level after long spinal fusion.

Boudriot et al. [2] showed a massive thoracic herniation caused by repeated adjacent instability of the thoracolumbar spine after lumbar fusion. In that case, it is believed that frequent motion at the physiological range of limits of the proximal segments after lumbar fusion might lead to an accelerated degenerative process.

In the other two cases [3, 4], junctional failure occurred in the thoracic disc level itself, without any failure of the upper instrumented vertebra and the supra-adjacent vertebra. According to Badra et al. [3], stress concentration at the proximal adjacent disc space between two stiff vertebrae led to acute disc failure.

Like the latter two herniation cases, our case had no apparent instability at the adjacent level until the herniation occurred. However, our case differs from the previously presented cases with respect to the fusion level and length.

Soh et al. [9] investigated the risk factors in adjacent segment degeneration after >5 years of follow-up after lumbar spinal fusion. They stated that there was a correlation between decreases in the fusion segment lordotic angle and the postoperative adjacent degenerative change; but sex, age, residential area, fusion method, number of fusion segments, and the degree of preoperative adjacent disc degeneration on MRI showed no significant relationship with it.

Meanwhile, Miyakoshi et al. [10] reported that although there was no contribution to the clinical results, all intervertebral disc heights adjacent to the fusion decreased in their 45 patients who underwent single-level posterior lumbar interbody fusion. Interestingly, the height of the adjacent disc and that of one level above it showed a statistically significant decrease 3 years after operation.

In our case, there was neither a lordotic angle change in the fusion segment nor proximal junctional instability until the herniation occurred. There was slight decrease in proximal adjacent posterior disc height but no change in adjacent vertebral body height until the herniation occurred. Lumbar MRI confirmed that the preoperative multiple severe disc degeneration already existed and that only the L2/3 disc was “intact,” and there was no apparent degeneration in the lumber level. According to Pfirrmann classification, L1/2, L3/4, and L4/5 discs were grade V before the fusion operation, meaning that they had already collapsed. Massive rupture of this intact disc might occur due to over loading of L2/3 between solid fused L3, 4, and 5 segments and fibrotic fused segments L1 and 2.

It remains controversial whether the spinal fusion should be extended to the upper level during the initial operation in cases such as these. However, it may be agreed that maximum preservation of the supraspinous and interspinous ligaments above the fusion level during the operation and careful watching after fusion operation are very important.

Here, we described the first case of adjacent lumbar disc herniation after lumbar spinal short fusion with acute paraplegia and urinary disturbance. The possibility of adjacent disc degeneration or disc hernias should always be considered after spinal fusion.
